# Point-of-care Ultrasound Identification of Hepatic Abscess in the Emergency Department

**DOI:** 10.5811/cpcem.1299

**Published:** 2023-04-18

**Authors:** Michael Blomquist, Taz Brinkerhoff, David Weech, Heesun Choi

**Affiliations:** * Rocky Vista University of Osteopathic Medicine, Parker, Colorado; † Kingman Regional Medical Center, Department of Emergency Medicine, Kingman, Arizona; ‡ University of California, Irvine, Department of Emergency Medicine, Orange, California

**Keywords:** Point-of-care ultrasound, hepatic abscess, pyogenic liver abscess

## Abstract

**Case Presentation:**

A 92-year-old female with past medical history of hypertension presented to the emergency department with pain in her right shoulder, right flank, and right upper quadrant of her abdomen. Point-of-care ultrasound (POCUS) and computed tomography imaging showed concerns for multiple large hepatic abscesses. Percutaneous drainage removed 240 millileters of purulent fluid that identified *Fusobacterium nucleatum*, a rare cause of pyogenic liver abscess.

**Discussion:**

Emergency physicians should keep hepatic abscess on their differential for right upper quadrant abdominal pain and can use POCUS for expeditious diagnosis.

## CASE PRESENTATION

**Image 1:**
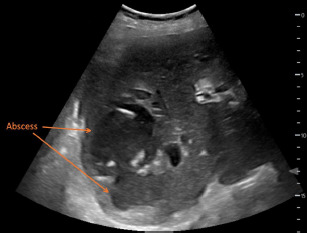
Soft tissue point-of-care ultrasound showing multiple, hypoechoic, loculated fluid collections within the parenchyma of the right lobe of the liver, indicative of hepatic abscesses.

A 92-year-old female with past medical history of hypertension presented to the emergency department with one week onset of pain in her right shoulder, right flank, and right upper quadrant of her abdomen. The patient’s initial presentation was consistent with a cholestatic pattern of gallbladder disease; however, further evaluation via point-of-care ultrasound (POCUS) of hepatobiliary structures revealed multiple large abscesses within the liver parenchyma (Image 1 & Video).

**Image 2:**
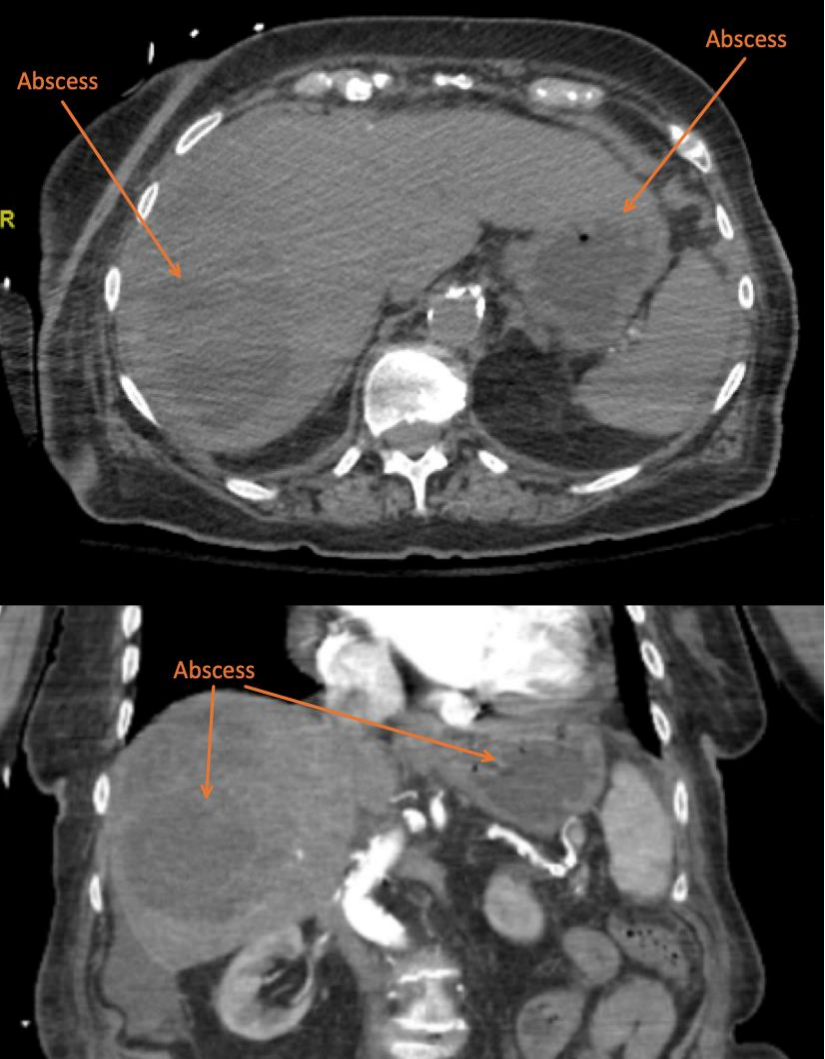
Computed tomography imaging with transverse (top) and coronal (bottom) plane views of the upper abdomen showing large septated fluid collection in the right hepatic lobe, most consistent with a large hepatic abscess, and additional fluid collection in the left hepatic lobe also consistent with hepatic abscess.

Computed tomography (CT) was obtained prior to labs due to concerning physical exam, which revealed large septated fluid collection in the right hepatic lobe most consistent with a large hepatic abscess and an additional fluid collection in the left hepatic lobe also consistent with hepatic abscess (Image 2).

## DISCUSSION

The initial POCUS findings were indicative for large hepatic abscess and significantly raised clinical concern for sepsis. In this case, POCUS was performed prior to lab work as was CT, which facilitated an expedited diagnosis of a severe, life-threatening disease. The patient was started on empiric antibiotic therapy and underwent percutaneous, ultrasound-guided drainage and drain placement that removed 240 of purulent fluid. During her admission she developed multisystem organ failure and was ultimately transitioned to hospice care.

Pyogenic liver abscesses are relatively uncommon, with an annual estimated incidence of 3.6 cases per 100,000 people in the United States, and have an estimated mortality rate of 5.6%.^1^ In North America, the most common cause of pyogenic liver abscess is direct spread from biliary infection; however, almost any bacterial infection of the gastrointestinal tract can undergo hematogenous spread to the liver via the portal circulation.^2^ The most common pathogens consistently identified from abscess cultures are *Escherichia coli*, *Klebsiella* species, and *Streptococcus* species.^1^ Interestingly, cultures from the abscess fluid drained from this patient identified *Fusobacterium nucleatum*, a rare cause of pyogenic liver abscess that is thought to be related to periodontal disease and sigmoid diverticulitis.^3^

Patients with hepatic abscess most commonly present with fevers and abdominal pain; however, symptoms can include a broad range of complaints such as nausea, vomiting, and weight loss. Jaundice may be the first and only clinical manifestation of the disease.^4^ The diagnosis of pyogenic liver abscess relies heavily on prompt imaging. Computed tomography is somewhat more sensitive for liver abscesses than ultrasound (approximately 95% vs 85%).^5^ If ultrasound was performed initially but did not demonstrate any abnormalities, CT should be performed if concern for a possible liver abscess or additional underlying pathology remains high.

*CPC-EM Capsule*

What do we already know about this clinical entity?

*Pyogenic liver abscess is rare yet its estimated mortality rate is 5.6%. The clinical presentation of pyogenic liver abscess is nonspecific which can cause delay of care. Performing POCUS early during the initial evaluation can provide narrow differentials. It is relatively easy to Identify large fluid collections in the liver as shown in the images*.

What is the major impact of the image(s)?

*Patient’s presentation was concerning for gallbladder pathology. However, POCUS finding indicated large septal fluid collection in the right hepatic lobe. This critical POCUS finding allowed physicians to deliver prompt treatment plans for the patient without a delay*.

How might this improve emergency medicine practice?

*Performing POCUS can provide faster, specific and accurate assessment and treatment plans on an elderly patient with undifferentiated abdomen pain. This can decreased financial burden to the patients, duration of ED stay, and ED wait time. treatment plans. Also providing specific labs and imaging studies can decreased financial burden to the patients, total hours of ED stay, and ED wait time*.

## Supplementary Information

Video Soft tissue point-of-care ultrasound showing multiple, hypoechoic, loculated fluid collections within the parenchyma of the right lobe of the liver, indicative of hepatic abscesses.
